# Competing metabolic strategies in a multilevel selection model

**DOI:** 10.1098/rsos.160544

**Published:** 2016-11-16

**Authors:** André Amado, Lenin Fernández, Weini Huang, Fernando F. Ferreira, Paulo R. A. Campos

**Affiliations:** 1 Evolutionary Dynamics Lab, Department of Physics, Federal University of Pernambuco, 50670-901 Recife, Pernambuco, Brazil; 2 Department of Evolutionary Theory, Max Planck Institute for Evolutionary Biology, August-Thienemann-Straße 2, 24306 Plön, Germany; 3 Center for Interdisciplinary Research on Complex Systems, University of São Paulo, 03828-000 São Paulo, Brazil

**Keywords:** metabolic pathways, evolutionary game theory, resource-based model

## Abstract

The evolutionary mechanisms of energy efficiency have been addressed. One important question is to understand how the optimized usage of energy can be selected in an evolutionary process, especially when the immediate advantage of gathering efficient individuals in an energetic context is not clear. We propose a model of two competing metabolic strategies differing in their resource usage, an efficient strain which converts resource into energy at high efficiency but displays a low rate of resource consumption, and an inefficient strain which consumes resource at a high rate but at low yield. We explore the dynamics in both well-mixed and structured populations. The selection for optimized energy usage is measured by the likelihood that an efficient strain can invade a population of inefficient strains. It is found that the parameter space at which the efficient strain can thrive in structured populations is always broader than observed in well-mixed populations.

## 1. Introduction

The handling of a common resource by individuals with different means to exploit the resource often gives rise to social dilemmas [1]. Here social dilemma is referred to as the situation in which individuals benefit from selfishness unless everyone chooses the same alternative. One of the well-known examples is observed when these features are related to how rapidly and how efficiently the resource is exploited. With the massive available data from experiments, especially in microbial populations, the social dilemma ensuing from resource competition has been more effectively addressed [2]. The conflict arises directly from the trade-off between growth rate and yield of resource exploitation. Empirical studies demonstrate that the trade-off between resource uptake and yield is commonplace in the microbial world [3–6]. The trade-off owes to biophysical limitations which prevent organisms from optimizing multiple traits simultaneously. Independent experiments have reported the existence of a negative correlation between rate and yield [3,7–9]. The existence of the trade-off for adenosine triphosphate (ATP)-producing reactions can be derived using arguments from irreversible thermodynamics [2,10].

Important insights have been gained regarding the understanding of energy efficiency at the cell standpoint. By energetic efficiency we mean the ability of cells to extract energy for a given amount of resource. It was shown that gene expression across the whole genome typically changes with growth rate [11]. This evidence naturally emerges from the assumption of occurrence of different trade-offs at the intracellular level [11,12], regardless of the metabolic pathway used to convert resource into ATP. The pathway choice dictates the speed and efficiency of growth. Heterotrophic organisms are good examples of the yield–rate trade-off. Their metabolism converts resource like glucose into energy in the form of ATP, mainly through two processes: fermentation and respiration [13,14]. Fermentation does not require oxygen and uses glucose as a reactant to produce ATP. However, it is considered to be inefficient because the end products still contain a great deal of chemical energy. On the other hand, in respiration the glucose undergoes complete oxidation. It is efficient because several ATPs are produced from a single glucose molecule [15]. A microorganism using fermentation depletes the resource faster than microorganisms using respiration [13–15]. However, respiration allows cells to make a better use of resource and to produce more offspring given the same amount of resource. In a population of cells using different metabolic processes, there will be competition between highly efficient cells (respiration) and inefficient cells (fermentation).

This competition among traits presenting distinct metabolic pathways is the concern within a broad evolutionary perspective. The physiological states of a cell play an important role. At the physiological level, previous investigations have tried to understand the underlying conditions that may trigger the switch from one mode of metabolism to another [6]. The most influential factor studied so far is the resource influx rate into the system. This problem has been addressed in the framework of spatially homogeneous environments [16], as in spatially structured environments of unicellular organisms [5,17]. The studies of the steady state of population dynamics were carried out either by writing down a set of coupled differential equations describing population density and resource dynamics or by directly mapping the problem into a Prisoner’s Dilemma game, thus requiring some arbitrary definition of a pay-off matrix in terms of the resource influx rate. Conclusions can then be drawn from the established results in the field of evolutionary game theory. According to those studies, competition always drives the strain with the lowest rate of ATP production to extinction in homogeneous environments [5]. On the other hand, when space is structured, the outcome of the system is determined by its free energy content (resource influx rate). If the resource is abundant, the strain using the pathway with high rate and lower yield dominates. If the resource is limited, the strain using the pathway with high yield but at lower growth rate dominates [5].

Despite the attempts to study this trade-off on structured populations, these works have focused on spatially structured populations [5,17]. While this provides a valuable approach to study biological structures like biofilms, it lacks a clear definition of group and group reproduction, essential features of multicellular organisms as they are usually defined in animals, plants and fungi [18]. In this sense, these models describe more a multi-organism community rather than a multicellular organism. It is known that multicellular life can appear without a biofilm-like phase, for example, through the experimental evolution of multicellularity [19]. Our approach does not depend explicitly on spatial structure, instead we focus on the group structure. This way, we are able to study multicellular structures that can reproduce as groups. It is also critical to understand which evolutionary force can turn these multicellular structures into evolutionary stable units.

This work aims to investigate the conditions under which the optimized handling of resource can prevail when selection acts at different levels of biological organization. As such, a model of structured populations with multiple groups is presented. We assume explicit competition among individuals for resource. Although resource competition occurs at the individual level, the groups are assumed to grow and split at rates which depend on composition, putting this model within the framework of multilevel selection. Therefore, a higher level of organization is formed and the group itself could be seen as a replicating entity, a premise for the establishment of a multicellular organism. The issue especially motivated as multicellularity is conjectured to have arisen almost concomitantly with the great oxygenation event, and thereby allowing the evolution of the respiration mode of cellular metabolism.

Here, the problem is mainly addressed within a stochastic context whereby an evolutionary invasion analysis is considered. We do extensive computer simulations, though analytical developments are also carried out in the special case of well-mixed populations. A great limitation of using deterministic approaches to study population dynamics [5] is that the neglected stochastic effects may be critical in the evolution of natural populations, especially in small populations, a very likely situation if the resource is not abundant. Although this problem has been mapped into a Prisoner’s Dilemma game with constant pay-offs [16,17], it is based on a simplified assumption of linear interactions among individuals. Instead, we avoid these limitations by making a thorough exploration of the cellular properties and measuring their effects on the population dynamics. This is certainly a gap in this literature, as pointed out by MacLean [2].

The paper is organized as follows. In §2, we present the details of our model and simulations. In §3, we derive the equilibrium solutions, perform an invasion analysis and explain the simulation results. In §4, we give a detailed discussion of our results.

## 2. Methods

### 2.1. Resource-based model description

We consider a structured population with groups. The population consists of individuals which use either of the two competing metabolic strategies: (i) efficient use of resource, denoted by *C*, at which an individual converts resource into ATP efficiently and thereby achieves a high yield and (ii) rapid metabolism, hereby denoted by *D*, with which an individual consumes resource at a high rate but converts it into ATP inefficiently.

**Figure 1.**
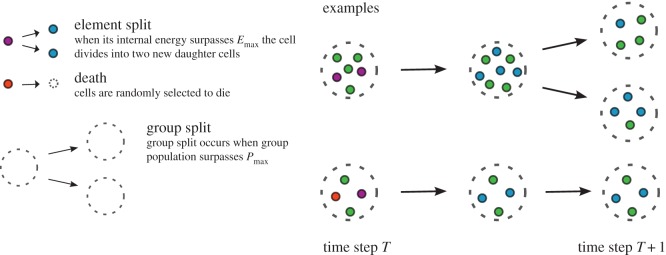
A graphical representation of the processes of group splitting, cell division and death.

The number of groups *N*_G_ and their local population sizes *P*_*i*_ (*i*=1,…,*N*_G_) vary over time. The influx of resource to the system (e.g. glucose) as well as how the resource is used are critical for the population dynamics. The population size increases or shrinks according to the amount of resource provided [20,21]. In every time step, individuals can produce new individuals and groups can split into new groups [22,23]. Each individual divides into two identical cells as soon as its internal energy storage *E*_*ij*_ (group index, *i*=1,…,*N*_G_, and individual index, *j*=1,…,*P*_*i*_) surpasses the energy threshold *E*_max_. Each daughter cell inherits half of the energy of the parental cell. Groups have a threshold size *P*_max_, above which a group will split into two with its members being evenly distributed between the two new groups. In every time step, each individual dies with a constant probability *ν*, i.e. in every time step and for each individual a random number x is drawn from a uniform distribution, x∈[0,1], and if x is smaller than *ν*, the individual dies. Both group splitting and the death process introduce stochasticity into the system. A group disappears when the number of individuals in that group shrinks to zero. The above-described dynamical rules are also illustrated in figure 1.

The amount of resource available to the population is constant and equal to *S* in every time step. We consider equipartition of resource among groups and thus the amount of resource per group is *S*_G_=*S*/*N*_G_. Note that the total number of groups *N*_G_ is a dynamical number. We break the complete process of an individual gaining energy into two stages: first, individuals compete for the resource; second, the caught resource is converted into internal energy (ATP). The assumption of these two stages is in accordance with mechanistic models of cells [11]: first, an enzyme transports the resource into the cell; then, a second set of enzymes metabolizes the resource into a generic form of energy, which includes ATP. The rates of resource import and of metabolism are distinct [11]. The metabolism of a cell is subject to multiple levels of regulation [24–26]. It is known that nutrients are used as substrates for growth but the nutritional state also supplies signals for the cell [27]. For example, cAMP (cyclic adenosine monophosphate) plays a central role in the regulation of the response of *Escherichia coli* to different nutritional states [28], and the *design* of the cell is an evolutionary choice that tunes it to act effectively within its biological niche. Note that our model does not refer to the resolution of processes at the intracellular level.

#### 2.1.1. Implementing the resource uptake

The resource is shared among the members of a group. As a strain type with rapid metabolism, *D*, has a higher rate of consumption, it seizes a larger portion of the resource available to the group compared with strains type *C*, characterized by a low consumption rate. The amount of resource per strain *D* in a given group *i* is estimated as

2.1 $$S_{D}^{i}(S_{G})=\frac{J_{D}^{S}(S_{G})}{J_{D}^{S}(S_{G})P_{D}^{i}+J_{C}^{S}(S_{G})P_{C}^{i}}S_{G},$$

where $P_{D}^{i}$ and $P_{C}^{i}$ are, respectively, the number of individuals for type *D* and *C* in group *i*, such that $P_{D}^{i}+P_{C}^{i}=P^{i}$ corresponds to the size of group *i*. The consumption rates for strain type *C* and *D*, $J_{C}^{S}(S_{G})$ and $J_{D}^{S}(S_{G})$, are functions of *S*_G_, the amount of resource per group. Similarly, the amount of resource per strain *C* is

2.2 $$S_{C}^{i}(S_{G})=\frac{J_{C}^{S}(S_{G})}{J_{D}^{S}(S_{G})P_{D}^{i}+J_{C}^{S}(S_{G})P_{C}^{i}}\;S_{G}.$$

Therefore, the total amount of resource for group *i* is $S_{D}^{i}(S_{G})P_{D}^{i}+S_{C}^{i}(S_{G})P_{C}^{i}=S_{G}$ as declared above.

#### 2.1.2. Implementing the conversion of resource into internal energy

The resource seized by the cell is used to increment its internal energy *E*_*ij*_, which is calculated as

2.3 $$\Delta E_{ij}=J_{C}^{\mathrm{ATP}}(S_{C}^{i})\Delta t\;\text{or}\;\Delta E_{ij}=J_{D}^{\mathrm{ATP}}(S_{D}^{i})\Delta t,$$

depending on the strain type, *C* or *D*. The functions $J_{C}^{\mathrm{ATP}}(S_{C}^{i})$ and $J_{D}^{\mathrm{ATP}}(S_{D}^{i})$ represent how efficiently the resource ($S_{C}^{i}$ or $S_{D}^{i}$) is transformed into energy (ATP) for each individual type. In a discrete-time model as assumed here, Δ*t*=1. The units of the defined variables/parameters are given in the electronic supplementary material.

### 2.2. Simulation procedure

#### 2.2.1. Statistical measurement

In a pool of organisms using resource inefficiently, it is very likely that an alternative metabolic pathway arises by mutation. It is important to evaluate the probability that a single individual of type *C* invades and finally takes over the whole population. The simulations start from an isogenic population of strains *D*, which later evolves to a stationary regime. At this steady state, the population size is determined by the population dynamics and the resource availability. For this reason, the system is said to be self-organized. Once the homogeneous population evolves to its stationary states, a single strain *C* is introduced to replace a randomly chosen cell from the population. From this time on, the fate of the mutant type *C* is observed, i.e. the simulation is tracked until the mutant gets lost or fixed.

The fixation probability is simply the fraction of independent runs at which the efficient strain gets fixed. Because the population size itself depends on the parameters of the model, it is more reasonable to use the relative fixation probability defined as the absolute fixation probability divided by 1/*N*_st_. Here *N*_st_ stands for the population size of strains *D* at the moment the efficient strain is introduced. Thus, 1/*N*_st_ corresponds to the fixation probability of a mutant under neutral selection. If the relative fixation probability is larger than 1, the mutant type is said to be selected for, while a probability smaller than 1 means the strain is counter-selected.

#### 2.2.2. Parametrization of the model

The rates of resource uptake and the rate of converting resource into energy appearing in equations (2.1) and (2.2) are defined as

2.4*a* $$\begin{array}{rl} J_{C}^{S}(S_{G}) & =A_{C}, \end{array}$$

2.4*b* $$\begin{array}{rl} J_{D}^{S}(S_{G}) & =A_{D}, \end{array}$$

2.4*c* $$\begin{array}{rl} J_{C}^{\mathrm{ATP}}(S_{i}^{C}) & =A_{C}^{\mathrm{ATP}}\left(1-\exp(-\alpha_{C}^{\mathrm{ATP}}S_{C}^{i})\right) \end{array}$$

2.4*d* $$\begin{array}{rl} \;\text{and}\;J_{D}^{\mathrm{ATP}}(S_{i}^{D}) & =A_{D}^{\mathrm{ATP}}\left(1-\exp(-\alpha_{D}^{\mathrm{ATP}}S_{D}^{i})\right). \end{array}$$

The *J*^ATP^ functions display a sigmoidal shape, thus saturating for large values of *S*, similar to Michaelis–Menten functions [5,11]. The effects of the rates on the population dynamics are extensively studied here. For most of the parameters, we perform a sweep over the parameter space that covers the physically meaningful regions. The maximum values for the rates are chosen in such way that the rate–yield trade-off exists. By definition, the efficient strain *C* is the one that transforms resources into internal energy efficiently at the expense of the consumption rate, while strain *D* metabolizes fast but at low yield. The parameters above follow some conditions:

(i) *A*_*D*_ must be larger than *A*_*C*_ (*A*_*D*_>*A*_*C*_), which ensures that type *D* always has a higher consumption rate for the same amount of resource. As naturally emerging in the forthcoming calculations, the relevant parameter is in fact the ratio between these two quantities, *ϵ*=*A*_*D*_/*A*_*C*_.
(ii) Another condition is $\Delta_{\mathrm{ATP}}=\alpha_{D}^{\mathrm{ATP}}/\alpha_{C}^{\mathrm{ATP}}<1$. Again, the ratio between the exponents in the functions (2.4*c*,*d*) is a key quantity in the process. This condition warrants that strain *D* is less effective than strain *C* by generating energy from a given amount of resource, unless a very large amount of resource is gathered to be converted into energy. This is ensured when the ratio $\Gamma_{\mathrm{ATP}}=A_{D}^{\mathrm{ATP}}/A_{C}^{\mathrm{ATP}}$ is larger than 1. This allows a fast metabolic pathway for *D* strains to produce more energy in the case where they capture a much higher amount of resource than the efficient strain. This possibility is particularly important as it is known that many cells can work as respiro-fermenting cells, concomitantly using the two alternative pathways of ATP production. Such respiro-fermentative metabolism is a typical mode of ATP production in unicellular eukaryotes like yeast [6,16]. In such a situation, the yield of the whole process is reduced as the cell drives more resource to be metabolized inefficiently, a habitual behaviour under the condition of plentiful resource. Of course, the situation $A_{D}^{\mathrm{ATP}}<A_{C}^{\mathrm{ATP}}$ can also be seen, but the opposite $A_{D}^{\mathrm{ATP}}>A_{C}^{\mathrm{ATP}}$ represents the worst scenario for the efficient strain. If it can thrive in such a case, it will naturally be favoured under less harsh conditions.

### 2.3. Social dilemma

Besides setting down the constraints on the biologically relevant parameter values, it is also important to find the region of the parameter space at which the rate-yield trade-off in fact produces a tragedy of the commons [1]. As commonly defined, the social dilemma exists if in a pairwise competition for a common resource strain *D* outcompetes strain *C* [2,5,29].

For a given amount of resource S*, the uptakes of resource by strains *C* and *D* are, respectively,

2.5 $$S_{C}=\frac{J_{C}^{S}}{J_{C}^{S}+J_{D}^{S}}S^{*}$$

and

2.6 $$S_{D}=\frac{J_{D}^{S}}{J_{C}^{S}+J_{D}^{S}}S^{*}.$$

The ratio between $J_{D}^{\mathrm{ATP}}$ and $J_{C}^{\mathrm{ATP}}$ provides us the relative advantage of strain *D* over strain *C* in a pairwise competition. From equations (2.4), we obtain that

2.7 $$r=\frac{J_{D}^{\mathrm{ATP}}}{J_{C}^{\mathrm{ATP}}}=\Gamma_{\mathrm{ATP}}\frac{1-\exp(-\alpha_{D}^{\mathrm{ATP}}S_{D})}{1-\exp(-\alpha_{C}^{\mathrm{ATP}}S_{C})}.$$

If this ratio is larger than 1, the social dilemma holds. This reasoning will be employed to map the set of parameter values where the social dilemma exists. As the ratio *r* depends on the amount of resource, in order to delimit the social dilemma region in a population of arbitrary size, we must use S*≃*S*/*N*, instead of using *S*, the total amount of resource. *N* denotes the population size. As observed from the simulations, the system evolves in such a way that *S*/*N* is always small at equilibrium. As we will see later, the limit of small *S*/*N* is useful to understand the results.

## 3. Results

We will start this section by presenting some analytical approximations for well-mixed populations. These calculations will later serve as a guidance to determine the situations in which simulation results match the theoretical prediction. They also help us to delimit the situations where phenomena driven by stochastic effects manifest, which are not captured by the analytical approach carried out here. As in the analytical approximations no population structure is assumed, the results will be particularly instructive to understand whether group structure is a driving force for the maintenance of the efficient strain.

### 3.1. Analytical results for well-mixed populations

#### 3.1.1. Equilibrium size of a population of strains with fast metabolism

Let us propose a discrete-time model for a well-mixed population with only cells of type *D*. More generally, the population size in time step *t*+1 can be written as

3.1 n(t+1)=n(t)+g(n(t),S)n(t)−νn(t),

where the function *g*(*n*(*t*),*S*) denotes the growth rate of strain *D*, which depends on the population size as well as on the availability of resource per time step, *S*. The death rate is denoted by *ν* (as a mathematical guide for the analytical development to be done, see [30]). In the above equation, it is implicitly assumed that *g*(*n*(*t*),*S*) is proportional to the amount of resource captured by the consumer. The conversion of resource into energy (directly translated as growth rate) occurs into two steps: uptake of resource and its posterior conversion into energy. As there is only one type of strain in a homogeneous population, the amount of resource per individual at time *t* is just *S*/*n*(*t*). Following equation (2.4d), the growth rate is equivalent to $g(n(t),S)=a_{D}[1-\exp(-\alpha_{D}^{\mathrm{ATP}}S/n(t))]$, where $a_{D}=A_{D}^{\mathrm{ATP}}/E_{\max}$, which is a necessary rescaling because individuals only replicate if their internal energies surpass the threshold *E*_max_. Plugging this expression into equation (3.1) we get

3.2 $$n(t+1)=n(t)+a_{D}\left[1-\exp\left(-\frac{\alpha_{D}^{\mathrm{ATP}}S}{n(t)}\right)\right]n(t)-\nu n(t).$$

The system is under equilibrium when $n(t+1)=n(t)=\hat{n}$ and has two equilibrium solutions, $\hat{n}=0$ and $\hat{n}=-\alpha_{D}^{\mathrm{ATP}}S/\ln(1-\nu /a_{D})$. Note that the second solution is only meaningful if *ν*<*a*_*D*_.

#### 3.1.2. Stability analysis

Next, we analyse the local stability of the two equilibrium solutions. By writing the dynamics of the discrete-time model as *n*(*t*+1)=*f*(*n*(*t*)), a solution, $n=\hat{n}$, is said to be stable when −1<λ<1, where $\lambda =(df/dn){|}_{n=\hat{n}}$. Note that the derivative of *f*(*n*(*t*)) with respect to *n* must be evaluated at the equilibrium. A value of |λ|<1 warrants that any small perturbation from the equilibrium solution will be damped, bringing the system back to equilibrium. Whenever |λ|>1, any disturbance drives the system away from the equilibrium point.

For the first equilibrium, $\hat{n}=0$, λ is evaluated as $\lambda =(df/dn){|}_{\hat{n}=0}=1-\nu +a_{D}$, and so the solution is stable when *ν*>*a*_*D*_, i.e. $\nu E_{\max}>A_{D}^{\mathrm{ATP}}$. On the other hand, the second equilibrium, $\hat{n}=-\alpha^{\mathrm{ATP}}S/\ln(1-\nu /a_{D})$, provides $\lambda =(df/dn){|}_{\hat{n}=-\alpha_{D}^{\mathrm{ATP}}\;S/\ln(1-\nu /a_{D})}=1+a_{D}(1-\nu /a_{D})\ln(1-\nu /a_{D})$. Because *ν*<*a*_*D*_ in the range of validity of the solution, the logarithm is always negative, meaning λ<1. Therefore, the second solution is always stable in the range it is valid.

### 3.2. Evolutionary invasion analysis

#### 3.2.1. Invasion by the efficient strain

A further step of our analytical calculation is to check under which conditions a population of strain with rapid growth can be invaded by a mutant that uses the resource more efficiently. The simulations start from an isogenic population of strain *D*. After the population reaches the stationary state, a single type *C* individual is introduced. The number of individuals of strains *D* and *C* over time, *n*_*D*_ and *n*_*C*_, are determined by the following set of equations:

3.3 $$n_{D}(t+1)=n_{D}(t)\{1+a_{D}[1-e^{-\alpha_{D}^{\mathrm{ATP}}J_{D}^{S}S/(J_{D}^{S}n_{D}+J_{C}^{S}n_{C})}]-\nu \}$$

and

3.4 $$n_{C}(t+1)=n_{C}(t)\{1+a_{C}[1-e^{-\alpha_{C}^{\mathrm{ATP}}J_{C}^{S}S/(J_{D}^{S}n_{D}+J_{C}^{S}n_{C})}]-\nu \},$$

where $a_{C}=A_{C}^{\mathrm{ATP}}/E_{\max}$. It is important to point out that as there are two distinct strains, the amount of resource captured per individual is no longer *S*/*n*(*t*) but obeys the resource partition as calculated by equations (2.1) and (2.2). The dynamics is driven by a density-dependent growth rate. One possible equilibrium of the above system is

3.5 $${\hat{n}}_{D}=-\frac{\alpha_{D}^{\mathrm{ATP}}S}{\ln(1-\nu /a_{D})}\;\text{and}\;{\hat{n}}_{C}=0.$$

This equilibrium corresponds to the non-zero equilibrium of the resident population of strain *D*, as shown in §i. As aforementioned, the system is self-organized in such a way that the population size is reflected by the supply of resource into the system and also determined by the metabolic properties of its individuals. A less efficient metabolism entails that the accumulation of internal energy is slower. In such case the system cannot sustain a large population size (electronic supplementary material, figure S1). It is worth mentioning that as the regime of small *ν*/*a*_*D*_ is considered, it ensues that taking this limit into equation (3.5) the *per capita* resource availability *S*/*n* is proportional to *ν*/*a*_*D*_ and hence also pretty small. This finding is always observed in our simulations. Next, we analyse the stability conditions of this equilibrium under the invasion of a small quantity of efficient strains. For multiple-variable models, the local stability of the equilibrium solutions is found by looking at the eigenvalues of the Jacobian matrix of the system.

After determining the eigenvalues and setting the conditions for stability (see the electronic supplementary material), it is possible to determine a relationship delimiting the parameter region at which the efficient strain can invade the resident population of type *D* cells. For fixed $A_{C}^{\mathrm{ATP}}$ and variable $A_{D}^{\mathrm{ATP}}$, we find that

3.6 $$\Gamma_{\mathrm{ATP}}=\frac{\nu /a_{C}}{1-{\left(1-\nu /a_{C}\right)}^{\Delta_{\mathrm{ATP}}\epsilon }},$$

where $\Delta_{\mathrm{ATP}}=\alpha_{D}^{\mathrm{ATP}}/\alpha_{C}^{\mathrm{ATP}}$ and *ϵ*=*A*_*D*_/*A*_*C*_, as previously defined. Equation (3.6) is used for the comparison with the isocline *P*_*fix*_=1, the relative fixation probability equal to 1, obtained from simulations. Therefore, our finding shows that the solution, equation (3.5), is stable against invasion above the curve (3.6), while the strain *C* can succeed to invade in the parameter space under the curve.

#### 3.2.2. Invasion by the inefficient strain

Now the opposite situation is considered—the conditions under which a mutant with rapid metabolism (type *D*) can invade a population dominated by cells of type *C*. The calculations are straightforward as calculations are similar as in §i. As the resident population is initially composed of cells type *C* only, its size at equilibrium is $n_{C}={\hat{n}}_{C}=-\alpha_{C}^{\mathrm{ATP}}S/\ln(1-\nu /a_{C})$, and $a_{C}=A_{C}^{\mathrm{ATP}}/E_{\max}$.

The Jacobian matrix is now evaluated at

3.7 $${\hat{n}}_{C}=-\frac{\alpha_{C}^{\mathrm{ATP}}S}{\ln(1-\nu /a_{C})}\;\text{and}\;{\hat{n}}_{D}=0$$

as the strain *D* is assumed to be rare. Likewise, a relation between *Γ*_ATP_ and Δ_ATP_ is obtained, and the curve now delimits the parameter region at which the efficient strain is stable against invasion by the inefficient strain. It is found that

3.8 $$\Gamma_{\mathrm{ATP}}=\frac{\nu /a_{C}}{1-{\left(1-\nu /a_{C}\right)}^{\Delta_{\mathrm{ATP}}\epsilon }},$$

which is exactly equal to equation (3.6). However, now the solution, equation (3.7), is stable under the curve. Thus, we conclude that the parameter region *Γ*_ATP_ versus Δ_ATP_, where the efficient strain is evolutionarily stable against invasion by the selfish strain *D*, coincides with the region at which it can, while rare, invade an isogenic population of type *D* individuals.

### 3.3. Simulation results

In this section, we present the simulation results of the evolutionary invasion analysis. In the electronic supplementary material, we also provide simulation results and plots of the population size of pure *D* strains at equilibrium. This is critical as the population size determines the strength of stochasticity on the dynamics of the system.

#### 3.3.1. The relative fixation probability of a single individual of the efficient strain

**Figure 2.**
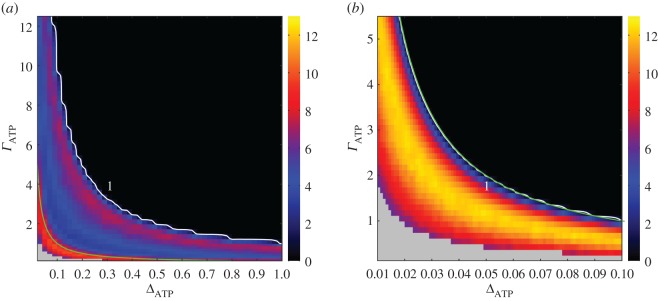
Relative fixation probability. In the plot, the relative fixation probability is shown in terms of $\Gamma_{\mathrm{ATP}}=A_{D}^{\mathrm{ATP}}/A_{C}^{\mathrm{ATP}}$ and $\Delta_{\mathrm{ATP}}=\alpha_{D}^{\mathrm{ATP}}/\alpha_{C}^{\mathrm{ATP}}$. (*a*) Structured populations and (*b*) homogeneous populations. The white thick lines denote the isocline where the relative fixation probability of a single cooperator is the same as the relative fixation probability of a random individual under neutral selection. Above the isocline the cooperative strategy is counter-selected (dark region), whereas under the line it is selected for. The green line corresponds to the line delimiting the social dilemma regime, obtained by making *r*=1 in equation (2.7). This line overlaps the lines given in equations (3.6) and (3.8). The grey region denotes that the defector population goes extinct before the cooperator is introduced. The other parameter values are resource amount *S*=25, death rate *ν*=0.01, group carrying capacity *P*_max_=10, internal energy for splitting *E*_max_=10 and *A*_*D*_=10. The data points corresponds to 40 distinct populations and for each population 10 000 independent runs were performed.

From the heat map in figure 2, we observe how the relative fixation probability is influenced by the ratios $\Gamma_{\mathrm{ATP}}=A_{D}^{\mathrm{ATP}}/A_{C}^{\mathrm{ATP}}$ and $\Delta_{\mathrm{ATP}}=\alpha_{D}^{\mathrm{ATP}}/\alpha_{C}^{\mathrm{ATP}}$. The ratio *ϵ*=*A*_*D*_/*A*_*C*_ is set at 10, which is a typical value, as empirically measured in populations of *Saccharomyces cerevisiae* [6]. Making the comparison with well-mixed populations, over a broader domain of the parameter space the efficient strain is selected for in the structured populations. Even when Δ_ATP_ is already close to 1, meaning that the yield of strain *D* is almost equivalent to strain *C*, the efficient strain can still persist. Overall, the domain at which the efficient strain is favoured in well-mixed populations is considerably restricted in comparison to structured populations. For instance, around Δ_ATP_=0.1 the efficient strain is already unlikely to invade and to fixate.

The curves corresponding to equations (3.6) and (3.8) are represented by the green line in figure 2*a*,*b*. The region below the green line portrays the domain at which the efficient strain can invade and replace a population of individuals of type *D*. Of course, the outcome of the dynamics is not deterministic, but it evidences a selective advantage for strain *C*. The white line is the isocline *P*_*fix*_=1 (the relative fixation probability is equal to 1). The line setting out the onset of the social dilemma corresponding to *r*=1 in equation (2.7) is also plotted. In well-mixed populations (figure 2*b*) the social dilemma line (*r*=1) overlaps with the green line, equations (3.6) and (3.8), and thence it is not perceivable in the plot. Indeed, in the limit *S*/*N* small, which always holds when *ν*/*a*_*D*_ is also small (see equation (3.5)), the two curves are essentially the same and become *Γ*_ATP_=1/Δ_ATP_*ϵ*. This observation leads us to conclude that in well-mixed populations the efficient strain is an effective intruder only in the domain where there is no dilemma. Note that the social dilemma line becomes independent of *S* in the limit *S*/*N* small.

On the other hand, in structured populations the efficient strain is selectively advantageous over a much larger set of the parameter space. The realm of the high yield strain goes further beyond the line setting out the social dilemma. The isocline *P*_fix_=1 is considerably shifted to the right. The region between the social dilemma line (green line) and the isocline *P*_fix_=1 is of special interest, as it shows the prominent role of structuring in promoting the fixation and maintenance of the strain exploiting the resource efficiently.

**Figure 3.**
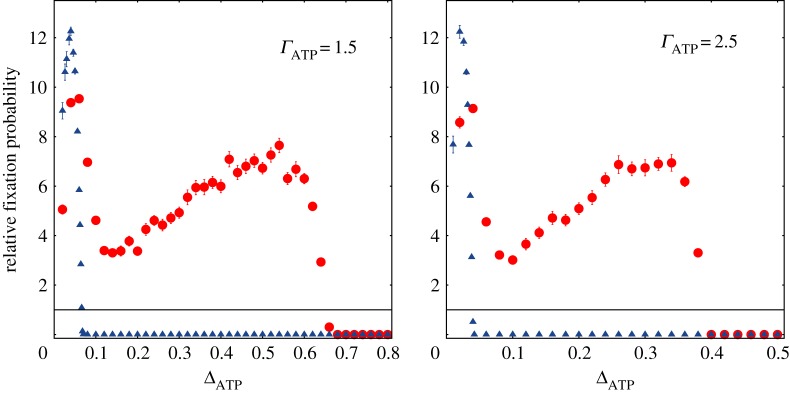
Relative fixation probability as a function of Δ_ATP_ for fixed values of *Γ*_ATP_. The blue triangles show the simulation outcomes for homogeneous populations, whereas the red dots for structured population. The data points correspond to 40 distinct populations and for each population 100 000 independent runs were performed. The black horizontal line denotes relative fixation probability 1. The parameter values are *S*=25, *ν*=0.01, *P*_max_=10, *E*_max_= 10, *A*_*D*_=10 and *A*_*C*_=1.

Looking at the heat map, it is notable that there exists a non-straightforward relationship between Δ_ATP_ and *Γ*_ATP_. At fixed *Γ*_ATP_ the relative fixation probability approaches a two-humped function of Δ_ATP_, being maximized at low and intermediate values of Δ_ATP_ (figure 3). In each of the panels of figure 3, *Γ*_ATP_ is kept constant while Δ_ATP_ varies. Well-mixed populations (blue triangles) display a smoother scenario and the relative fixation probability is now a one-humped function of Δ_ATP_. The point at which the relative probability is maximized depends on *Γ*_ATP_, being shifted towards higher values of the ratio Δ_ATP_ as *Γ*_ATP_ decreases. In well-mixed populations the selective advantage of strain *C* over strain *D* is confined to the region where there is no dilemma.

Yet, in structured populations the range of Δ_ATP_ in the plot embodies three distinct regions: the region at which there is no dilemma, a second region where the dilemma exists and the efficient strain has a selective advantage, and a third one at which the dilemma still holds but the efficient strain is no longer able to persist. The first peak occurs at small Δ_ATP_ as observed in well-mixed populations, but now there is a second peak that lies at the range of intermediate values of Δ_ATP_. The first peak is easily understood as it lies in the region of no social dilemma and the efficient strain already has a selective advantage. In this region, the ratio *r*, as defined in equation (2.7), is greater than 1. In fact, the advantage of the efficient strain over the inefficient one is observed in both structured and unstructured populations, emphasizing that cooperators are always favoured in this regime.

The second peak is more difficult to analyse, as we have the coupling between the dynamics of group formation and the within-group dynamics. At a first look, it may seem counterintuitive that the relative fixation probability increases with defector efficiency at intermediate values of Δ_ATP_. For explaining this result, we need to take into account how both local competition for resource and group expansion are affected. On the one hand, a more efficient defector (larger Δ_ATP_) leads to an increased strength of local competition for resource as experienced by the cooperator; on the other hand, it also allows a faster expansion of the group containing the invading cooperator, thus favouring group division and the spreading of the cooperative trait. Thus, the net advantage of the efficient strain comes from the net outcome of these two competing mechanisms. At very large values of Δ_ATP_, the local competition as experienced by the efficient strain is so strong, in such way it cannot reproduce inside the group, and it outweights the advantage brought about by the group expansion.

**Figure 4.**
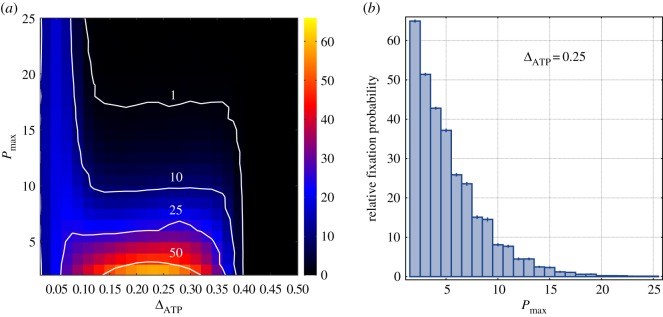
The strength of stochasticity. (*a*) The relative fixation probability in terms of the carrying capacity *P*_max_ and the ratio Δ_ATP_. Panel (*b*) represents how the relative fixation probability changes with *P*_max_ in a given Δ_ATP_. The thick white lines correspond to isoclines. The parameter values are *S*=25, *ν*=0.01, *E*_max_=10, *A*_*D*_=5 and *Γ*_ATP_=2.5. The data points correspond to 40 distinct populations and for each population 100 000 independent runs were performed.

Next, we study how the relative fixation probability changes with the group carrying capacity, *P*_max_. This enables one to assess the strength of stochasticity on the population dynamics. Previous studies have claimed that in multilevel selection the likelihood of invasion and posterior fixation of cooperative traits are particularly enhanced for small group sizes [23], evidencing the importance of stochasticity in the dynamics [31]. Similar behaviour was observed in a resource-based model of pairwise interactions within the multilevel selection framework [32]. The contour map in figure 4*a* displays the relative fixation probability for different values of *P*_max_ and different values of Δ_ATP_. The lighter colours in the bottom of the plot evince that the efficient strain *C* is significantly favoured as smaller group size is considered. For extremely small group sizes, like *P*_max_=5, the relative fixation probability varies enormously with the ratio Δ_ATP_, being maximized at intermediate values. In such small groups, the relative fixation probability can be 60-fold higher than the relative fixation probability of a neutral trait. This occurs because as smaller groups are considered there exists a greater chance of formation of uniform groups (comprised only cooperators) during the formation of new groups at an early stage of the introduction of the cooperative trait. Once a uniform group is produced, the cooperators are free of the exploitation of the defectors and the group enjoys a higher chance of fixation. At large values of *P*_max_, instead of a pronounced change with Δ_ATP_, the probability is roughly constant at a broad interval range of Δ_ATP_, as inferred from the isoclines. Of particular interest is the isocline delimiting the onset of the phase at which the efficient strain is favoured. From the isocline *P*_fix_=1, one deduces that for Δ_ATP_ between 0.1 and 0.4 there is a well-defined critical value for the group carrying capacity, *P*_max,c_≃17, beyond which the efficient strain becomes counter-selected. Although as the efficiency of the strain *D* is further reduced (small Δ_ATP_), the critical group size *P*_max,c_ increases again. For the sake of completeness, figure 4*b* shows that the relative fixation probability is a well-behaved monotonic decreasing function of *P*_max_ under fixed Δ_ATP_.

**Figure 5.**
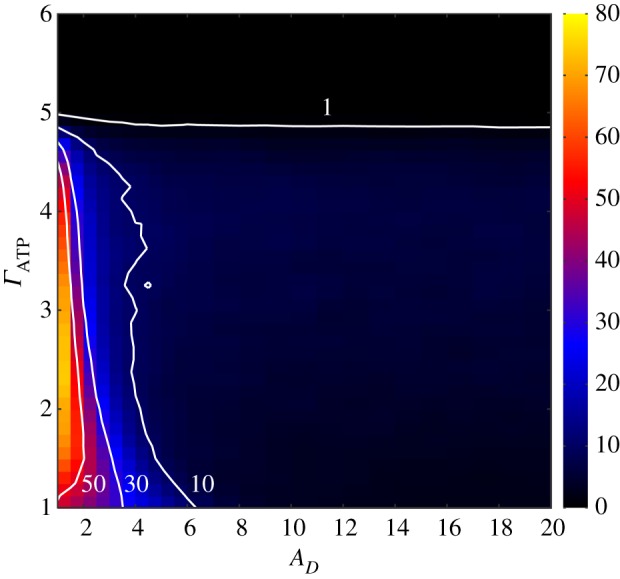
The effect of the consumption rate. Heat map of the relative fixation probability in terms of the ratio *Γ*_ATP_ and the consumption rate of defectors *A*_*D*_. The parameter values are *S*=25, *ν*=0.01, *P*_max_=10, *E*_max_=10 and Δ_ATP_=0.2. The thick white lines correspond to isoclines. The data points correspond to an average over 10 distinct populations and for each population 10 000 independent runs were performed.

How the consumption rate affects the fate of the efficient strain with a low frequency is studied in figure 5. A contour plot in terms of *Γ*_ATP_ and *A*_*D*_ is presented. The consumption rate is naturally a relevant part in the process as the amount of energy added to the individual’s internal energy is bounded to the amount of resource caught, which by its turn also influences other individuals’ performance due to competition. The selfish strategy has a twofold benefit when both *A*_*D*_ and *Γ*_ATP_ are kept high. First, in the competition for the resource, a high *A*_*D*_ means a big advantage in the resource uptake and subsequently in the process of converting resource into energy, where the advantage is determined by its efficiency (figure 5). As illustrated in the plot, the right corner is dark-coloured meaning that the efficient strain is clearly counter-selected. We also see that *Γ*_ATP_ has a more influential role than *A*_*D*_. At *A*_*D*_≃5, a further increase of the variable does not lead to any substantial variation of the relative fixation probability.

### 3.4. Enabling migration between groups

As aforementioned, increasing the group carrying capacity $P_{\max}$ will reduce the strength of stochasticity and thus creating a less favourable environment for the promotion of the efficient usage of resource. Here, we present the effects of an alternative natural force, migration between groups. At each time step every individual has a probability *m* to leave its original group and move to a randomly chosen group in the population. This is known in ecological studies as the island model of stochastic migration [33]. By allowing migration to happen, the effective population size can change and in the limit of high migration rates it is expected that the structured population behaves similar to its well-mixed counterpart.

**Figure 6.**
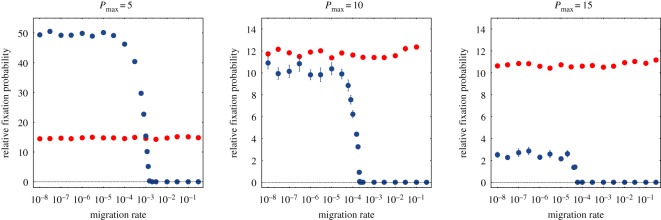
Effect of migration on the relative fixation probability. The relative fixation probability is plotted as a function of the migration rate *m* for three distinct values of carrying capacity *P*_max_. The parameter values are *S*=25, *ν*=0.01, *E*_max_=10, *A*_*D*_=10, *A*_*C*_=1, *Γ*_ATP_=1.5 and Δ_ATP_=0.5 (blue points) and Δ_ATP_=0.04 (red points).

The effect of the migration rate on the relative fixation probability can be seen in figure 6. Different situations are considered in the face of our previous knowledge about the system. Distinct values of Δ_ATP_ and of carrying capacity *P*_max_ are considered. At Δ_ATP_=0.5 (blue points) the efficient strain is selected against in well-mixed populations but not in structured populations. At Δ_ATP_=0.04 (red points), the efficient strain is favoured in both scenarios. In the latter situation, migration does not affect the outcome and the relative fixation probability is invariant.

Important information about the role of migration is gathered from the analysis of the case Δ_ATP_=0.5. Starting from small migration rates, which means the groups are more isolated, the relative fixation probability remains roughly constant over a wide range of migration rates, and then sharply goes to zero when the migration rate increases further, signifying less isolation between groups. The first plateau coincides with the value of the relative fixation probability in our structured population model, while after the transition the situation resembles well-mixed populations at which the efficient strain can no longer thrive.

## 4. Discussion

We have studied the evolutionary dynamics of a population with two strains under a trade-off between rate and yield. The two strains with distinct metabolic pathways compete under a scenario of limited resource. This problem has long been debated within the framework of evolution of cooperation, as the strain that uses resource efficiently is described as a cooperator, while the strain with high uptake rate, at the expense and degradation of its own environment, is said to display a defecting behaviour [5,34]. The trade-off between high rate and yield occurs due to biophysical restraints. Experimental results show a clear negative correlation between resource uptake and efficiency of conversion of resource into energy [3,5,35]. The existence of such trade-offs in heterotrophic microorganisms, like bacteria and yeast, and the fact that the efficient manipulation of resource was made possible with the appearance of the metabolic pathway that makes use of oxygen, the so-called respirers, have motivated a large body of studies. A clear motivation is the issue related to the major transition from single cells towards multicellular forms of life [36,37]. Multicellularity can thus be thought of as the integration of smaller units to build up a more complex machinery.

Here we proposed an explicit resource-based model in which competing metabolic strategies are assumed. We studied the conditions under which the cooperative strain with high yield metabolism can invade and outcompete a resident population of selfish strains. By investigating structured and well-mixed populations, we understand better whether the existence of group structure is a required condition for the maintenance of efficient metabolism pathways.

Our results show that in well-mixed populations, the efficient strain is only viable in a subset of the parameter space corresponding to the region where there is no social dilemma. In other words, the efficient strain can only invade a population of inefficient strains when it is rare, under the condition it is already a strong competitor in a pairwise competition with its opponent. The lines delimiting the social dilemma region and the isocline *P*_fix_=1 overlap. These lines also delimit the region of stability of the two competing metabolic strategies. Together, these results suggest that a complete mixing of cells creates an unfavourable scenario for the appearance and subsequent maintenance of an efficient mode of metabolism.

On the other hand, in structured populations a more favourable scenario is presented. Over a broad domain of the parameter space, which goes further beyond the boundaries set down by the social dilemma and the invasion analysis, the efficient strain is selected for. This result highlights the role of structuring, also corroborated by the study of migration effects on the dynamics of fixation of the efficient strain. As migration is reduced and so isolation increases, an abrupt augmentation of the relative fixation probability of the efficient strain is observed. Although local competition among relatives may increase in structured populations [38,39], it does not mitigate the benefits of increased stochasticity in the model. More evidence of this finding is obtained by checking the dependence of the relative fixation probability on the carrying capacity $P_{\max}$, whereby it is demonstrated that the efficient strain faces an even more favourable environment when smaller groups are assumed.

One question remains: which mechanisms can relieve the effect of increased competition in small groups? Local competition for resource is attenuated in the structured populations as groups are elastic and their sizes can vary between fission events. This is consistent with previous findings [39,40]. Although a larger group means less resource available per individual, there is no competition for space. Once one individual reproduces it does not necessarily lead to the removal of any interactant. Additionally, a group containing more efficient individuals has as advantage, as a larger amount of resource will be available and the net amount of energy converted in the process can be larger. This allows the group to reach the carrying capacity $P_{\max}$ faster. The third mechanism is the process of group split, which produces a twofold benefit: first by reducing local competition because after division the number of cells per group drops; second, if the number of efficient cells that occupy a given group increases, so does the chance that a newborn group is only filled by efficient strains, thus helping to perpetuate the trait. In summary, three premises of the model—group expansion, overlapping generations and group split—contribute to attenuate local competition.

The observation of a critical value of *P*_max_ imposes a threshold for the level of finiteness to act effectively. The group split is a key premise of the model. In a recent work, Ratcliff *et al.* [19] designed an experiment to understand the evolution of complex multicellular organisms from unicellular ancestors. In the experiment, a population of yeast (*S. cerevisiae*) was artificially selected for multicellularity by growing in nutrient-rich medium. Serial transfers of the bottom fraction of the liquid were performed, and cells collected together with the liquid formed the next generation. After a few transfers, they already observed that populations were dominated by snowflake-shaped multicellular clusters. Their witness of the occurrence of cluster reproduction is a basic premise to settle down life in its multicellular form. The authors ascertained that the daughter clusters were produced as multicellular propagules rather than mass dissolution of parental clusters. They also observed the existence of a threshold for the minimum size of daughter clusters [19]. In our viewpoint, the findings in the experiment of Ratcliff *et al.* are in line with the assumptions of the group dynamics of the proposed model in this work.

As conjectured, the first step in the evolutionary transition from single-celled to multicellular organisms most likely involved the evolution of undifferentiated multicellularity [36]. Our results show that it is very unlikely that the efficient mode of ATP production becomes widespread in a scenario of global interaction and competition (well-mixed populations). Even though efficient strains can arise through mutation, the maintenance of those strains in a well-mixed population would be very improbable as they could be easily invaded by strains with higher growth rate in spite of the low efficient metabolism. Alternatively, the appearance and subsequent growth of efficient strains in frequency must come together with the emergence of a mechanism that prevents dividing cells from complete separation enabling clusters through parent–offspring adhesion [19]. Collecting efficient feeders into a group, whether they are of the same kin or not, would benefit them equally well.

## Supplementary Material

Supporting Information - The supplementary material provides detail about the stability analysis of the solutions

## Data accessibility

Our C++ codes are available at Dryad Repository in http://dx.doi.org/10.5061/dryad.q6784 [41].
