# Supplementary material for: Competing metabolic strategies in a multilevel selection model
Source: R Soc Open Sci. 2016 Nov 16;3(11):160544. doi: 10.1098/rsos.160544 (PMC5180140; doi:10.1098/rsos.160544)
Supplement: Supporting Information - The supplementary material provides detail about the stability analysis of the solutions [file rsos160544supp1.pdf]

Electronic Supplementary Material from "Competing metabolic strategies in a multilevel selection model"

This electronic supplementary material provides detailed analytical calculations for the equilibria and stability analysis for well-mixed populations. Additionally, it provides the parameters summary with their description and units.

1 The Jacobian matrix

Here we calculate the eigenvalues of the Jacobian matrix of the discrete model. If the absolute values of the eigenvalues are smaller than one then the solution in Section 3.2.1 is stable. If we write the set of equations (10) of our two-variable model as $n_D(t+1) = f_D(n_D, n_C)$ and $n_C(t+1) = f_C(n_D, n_C)$, the Jacobian matrix is defined as

$$J = \begin{pmatrix} \frac{\partial f_D}{\partial n_D} & \frac{\partial f_D}{\partial n_C} \\ \frac{\partial f_C}{\partial n_D} & \frac{\partial f_C}{\partial n_C} \end{pmatrix}.$$

The Jacobian matrix must be evaluated at the equilibrium of interest, i.e. $\hat{n}_C = 0$ and $\hat{n}_D = -\frac{\alpha_D^{ATP} S}{\ln(1-\nu/a_D)}$. After some algebra, the coefficients of the matrix at the equilibrium of interest are determined

$$J = \begin{pmatrix} 1 + a_D(1-\nu/a_D)\ln(1-\nu/a_D) & \frac{a_D}{\epsilon}(1-\nu/a_D)\ln(1-\nu/a_D) \\ 0 & 1-\nu+a_C\left[1-(1-\nu/a)^{\frac{1}{\Delta_{ATP}\epsilon}}\right] \end{pmatrix},$$

where $\Delta_{ATP} = \frac{\alpha_D^{ATP}}{\alpha_C^{ATP}}$ and $\epsilon = \frac{A_D}{A_C}$. The above matrix is upper diagonal which means that the eigenvalues of the Jacobian are simply the diagonal coefficients. Because $\nu < a_D$ for the referred equilibrium, the element J_{11}

is always smaller than one and positive regardless the values of ν and a_D. Note that the logarithmic term is always negative. Therefore, the stability of the solution is uniquely settled by the element J_{22}. Of particular interest is the point at which the equilibrium becomes unstable which occurs when J_{22} equals one. Making J_{22} equal to one a relation between Γ_{ATP} and Δ_{ATP} is obtained.

The same analysis is done in Section 3.2.2, but instead the solution $\hat{n}_D = 0$ and $\hat{n}_C = -\frac{\alpha_C^{ATP} S}{\ln(1-\nu/a_C)}$ is considered.

2 Coexistence solution

The system of equations (10) also admits a solution where the two strains coexist. By rearranging the set of equations we verify that the coexistence solution obeys the following set of equations:

$$n_D\epsilon + n_C = -\frac{\alpha_D^{ATP} S\epsilon}{\ln(1-\nu/a_D)} \tag{SI 1}$$

$$n_D\epsilon + n_C = -\frac{\alpha_C^{ATP} S}{\ln(1-\nu/a_C)}, \tag{SI 2}$$

showing that there is an indeterminacy, and the coexistence solution only exists when $\Delta_{ATP} = \frac{1}{\epsilon}\frac{\ln(1-\nu/a_D)}{\ln(1-\nu/a_C)}$. Actually Eq. (SI 1) describes a family of solutions. Of course, the range of values at which n_D and n_C are meaningful are $n_C \in [0, \mathcal{N}]$ and $n_D \in [0, \mathcal{N}]$, where $\mathcal{N} = -\frac{\alpha_C^{ATP} S}{\ln(1-\nu/a_C)}$ with the constraint that $n_D\epsilon + n_C = -\frac{\alpha_C^{ATP} S}{\ln(1-\nu/a_C)}$. Note that when $n_C = 0$ we recover the solution $n_D = -\frac{\alpha_D^{ATP} S}{\ln(1-\nu/a_D)}$, whereas if $n_D = 0$ we recover the solution $n_C = -\frac{\alpha_C^{ATP} S}{\ln(1-\nu/a_C)}$. By determining the Jacobian of the system at equilibrium and hence calculating its corresponding eigenvalues we check whether the coexistence solution is stable or not. With the help of *mathematica*, we performed a numerical computation of the eigenvalues for different set of parameters by varying all the range of acceptable values of n_D (since n_C is written as a function of n_D). As expected in this situation, one of the eigenvalues is equal to one, which means that any disturbance in the same direction as the line drives the system to the new values of (n_D, n_C). This is called marginal stability as found in a simple predator-prey model studied by Lotka and Volterra (Parker and Kamenev 2010). The stability of the family of solutions will be dictated by the second eigenvalue. For the set of parameter values used in our simulations the second eigenvalue is slightly smaller than one, meaning that any transverse disturbance is damped by the system, thus

warranting stability of the family of solution in Eq. (SI 1). It is important to highlight that the coexistence solution only holds for $\Delta_{ATP} = \frac{1}{\epsilon}\frac{\ln(1-\nu/a_D)}{\ln(1-\nu/a_C)}$, which describes a line in the diagram Δ_{ATP} versus Γ_{ATP}.

In addition, from Eq. (10) we also notice that $\hat{n}_D = 0$ and $\hat{n}_C = 0$ are also equilibrium solutions, where both types go extinct. The solution is only stable when $\nu > a_D$ and $\nu > a_C$.

The coexistence of the two strains can not be observed in the simulations as the family of solutions embodies the absorbing states $n_C = 0$ and $n_D = 0$. As stochasticity plays a key role in finite populations, in a finite time the system will eventually reach one of these boundaries. Since we always start from $n_C = 1$, the system will probably evolve to $n_C = 0$ in a short time.

3 Population size at equilibrium

Here we show how the stationary population size of individuals type D (before introducing a single strain C), $N_{\rm st}$, depends on the set of parameters. From this we gain some insight about the strength of stochasticity. As intuitively expected, a linear relation between population size and the influx of resource into the system is observed (data not shown). The linearity is not followed when studying the dependence of the population size on the other parameters.

In Figure S1 we show the population size in terms of $\Gamma_{ATP} = A_D^{ATP}/A_C^{ATP}$ and $\Delta_{ATP} = \alpha_D^{ATP}/\alpha_C^{ATP}$, for both structured (left panel) and panmictic populations (middle panel). The values of Γ_{ATP} and Δ_{ATP} are varied by keeping $A_C^{ATP} = 2$ and $\alpha_C^{ATP} = 0.5$ and changing A_D^{ATP} and α_D^{ATP}, respectively. The range of values of Γ_{ATP} and Δ_{ATP} are, as argued before, those that are meaningful to the problem here addressed. Please note that a logarithmic scale is adopted for the color gradation in Fig. S1. The range of $\Delta_{ATP} = \alpha_D^{ATP}/\alpha_C^{ATP}$ in which population size is shown for the well-mixed populations is much smaller than the range displayed for structured population. Outside of this range the population already falls in the regime where the efficient strain is counter-selected - as can be seen in Fig. 2 (Main Text). The right panel of Fig. S1 shows the equilibrium solution for the discrete-time model of a well-mixed population, given by $\hat{n} = -\frac{\alpha_D^{ATP} S}{\ln(1-\nu\frac{E_{max}}{A_D^{ATP}})}$, as derived in Section 3.1.1. The grey region denotes the extinction of the population, where the above solution becomes unstable, while the solution $\hat{n} = 0$ becomes stable. The plot evinces a very good agreement between the theoretical prediction and simulations. The simulation results for a well-mixed population display a larger grey area in comparison to the theoretical prediction, which owes to stochastic effects, which are not captured by the time-discrete model. In the

right panel, the population size is too small — around five or less individuals — being prone to extinction.

Especially, in structured populations the stationary population size N_{st} of a pure population of strain D can vary from less than 100 individuals (small Γ_{ATP} and Δ_{ATP}) to nearly $5,000$ individuals (large Γ_{ATP} and Δ_{ATP}). The wide range of the population size at equilibrium explains why we should adopt a relative measurement of fixation probability since the strength of drift, namely $1/N_{\text{st}}$, is variable under the change of the parameter values.

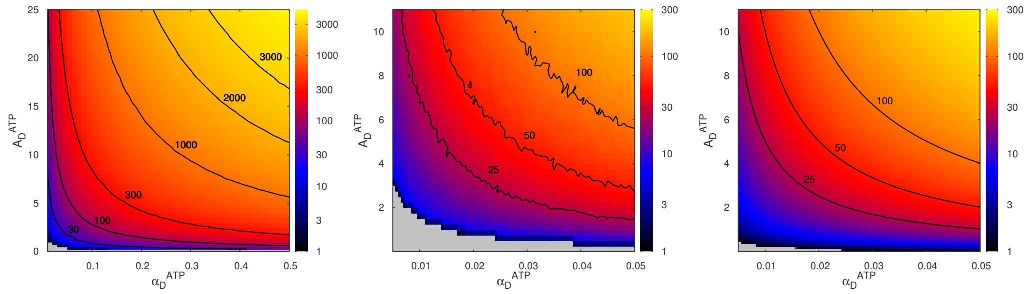

Figure S1: Stationary population size of defectors. The figure is a heat map of the population size of defectors (just before the introduction of a single cooperator) in terms of A_D^{ATP} and α_D^{ATP}. Left panel: simulation results for structured populations, middle panel: simulation results for well-mixed populations and right panel: theoretical prediction, $n_D = -\frac{\alpha_D^{ATP} S}{\ln(1-\nu E_{\max}/A_D^{ATP})}$. The other parameter values are influx rate of resource $S = 25$, death rate $\nu = 0.01$, group carrying capacity $P_{\max} = 10$, internal energy for one individual to split to two individuals $E_{\max} = 10$, and $A_D = 10$. The simulation data points correspond to 40 distinct populations and for each population $10,000$ independent runs were performed. The black lines denote isoclines, along which the population size is a constant (corresponding values indicated). The grey region denotes a region where the defector population is not sustainable and goes to extinction (before inserting the cooperator).

4 Parameters summary

For the sake of clarity we summarise the parameters of the model, including a short description and units. We use as basic units the time step, resource and energy.

Parameter	Interpretation	Units
ν	death rate	time-step^{-1}
S	total available resource	resource
A_C	rate of resource capture of C	resource/time-step
A_D	rate of resource capture of D	resource/time-step
A_C^{ATP}	maximum rate of resource processing of C	energy/time-step
A_D^{ATP}	maximum rate of resource processing of D	energy/time-step
α_D^{ATP}	efficiency of D in resource processing	resource^{-1}
α_C^{ATP}	efficiency of C in resource processing	resource^{-1}
E_{max}	energy threshold for cell split	energy
P_{max}	threshold size for group split	dimensionless
m	migration rate (probability of each cell to migrate to a random group per time-step)	time-step^{-1}

Also we include information about some derived quantities that are important to analyse the results, despite not being parameters of the model.

Quantity	Definition	Interpretation	Units
S_G	S/N_{groups}	resource available per group	resource
J_C^S	A_C	resource uptake of C	resource/time-step
J_D^S	A_D	resource uptake of D	resource/time-step
J_C^{ATP}	(see eq. 4)	rate of resource processing of C	energy/time-step
J_D^{ATP}	(see eq. 4)	rate of resource processing of D	energy/time-step
ϵ	A_D/A_C	fraction of the resource acquired by D in a pairwise interaction	dimensionless
Δ_{ATP}	α_D/α_C	relative efficiency of D and C	dimensionless
Γ_{ATP}	A_D^{ATP}/A_C^{ATP}	ratio between the maximum rates of resource processing of D and C	dimensionless
r	J_D^{ATP}/J_C^{ATP}	relative advantage of D over C in a pairwise interaction	dimensionless
a_D	A_D^{ATP}/E_{max}	maximum growth rate of the D	time-step^{-1}
a_C	A_C^{ATP}/E_{max}	maximum growth rate of the C	time-step^{-1}
ΔE_{ij}	$J^{ATP}\Delta t$	internal energy increase	energy

References

Parker, M. and A. Kamenev (2010). Mean extinction time in predator-prey model. *Journal of Statistical Physics 141*(2), 201–216.
